# Determining the New Norm Elements in Emergency Departments in Malaysia During a Pandemic: A Fuzzy Delphi Method

**DOI:** 10.21315/mjms2024.31.5.17

**Published:** 2024-10-08

**Authors:** Jivanya Raj Selvaraju, Nik Ab. Rahman Nik Hisamuddin

**Affiliations:** 1 Ministry of Health Malaysia, Federal Government Administrative Centre, Putrajaya, Malaysia; 2 Department of Emergency Medicine, School of Medical Sciences, Universiti Sains Malaysia, Kelantan, Malaysia; 3 Hospital Universiti Sains Malaysia, Universiti Sains Malaysia, Kelantan, Malaysia

**Keywords:** new norms, emergency departments, COVID-19, pandemic, Fuzzy Delphi

## Abstract

**Background:**

Emergency departments (EDs) have had to cope with various pandemics, such as HIN1, Ebola and the currently ongoing COVID-19. This study aimed to determine the elements of the new norm that has introduced changes into healthcare systems, particularly EDs, during the COVID-19 pandemic and to obtain consensus from the experts, the Emergency physicians in EDs across Malaysia. No previous study has been conducted on this topic.

**Methods:**

This study used the fuzzy Delphi method (FDM) to acquire expert consensus. There were two phases in this study. The first involved extracting the elements to be assessed by the selected experts from a literature review. Three major domains were considered: infrastructure, governance and human resources. A total of 35 items were identified and divided into the three domains. In the second phase, the selected items were sent to a group of 15 ED physicians, who were asked to rate the items on a Likert-type scale. The data were then analysed using FDM.

**Results:**

A total of 35 items were identified as possible new norms from a literature search for the three domains (governance, infrastructure and human resources). The first step of the FDM analysis showed that 9 out of the 35 items did not fulfil the initial requirement of the FDM, since the threshold value (*d*) must be lower than 0.2 (*d* </ 0.2). In meeting the second condition for the FDM, 25 out of the 35 items had an expert consensus of > 75%. Regarding the third requirement for FDM, only 1 out of the 35 items did not meet the criteria of an average fuzzy number (*A* value) of > 0.5. Finally, 25 items fulfilled all three requirements of FDM, so these were retained and the remaining 10 items were discarded.

**Conclusion:**

The FDM used in this study had identified 25 items achieved the required level of agreement by the chosen experts. The results of this study can be used to guide EDs in Malaysia to utilise the new norms items in mitigating major outbreak affecting the ED services.

## Introduction

COVID-19 is a global infectious disease, with its first outbreak reported in Wuhan, China. COVID-19 infection has caused millions of deaths worldwide, leading the World Health Organization (WHO) to declare a pandemic. The rapid spread of the pandemic is a ramification of human movement and global communication. To date, COVID-19 has remained active, costing the world in various ways, including human lives, the economy, travel and healthcare capacity (1).

The COVID-19 pandemic has impacted and exposed the limitations of healthcare systems, especially in their management and delivery of treatment. In particular, it has challenged the global healthcare system’s capacity to prepare for and respond to infectious diseases. The success of healthcare systems in managing the pandemic has largely depended on their organisation, coordination and governance. Therefore, the lessons learned during the COVID-19 pandemic can strengthen healthcare systems’ preparedness to respond and combat emerging infectious diseases in the future. Coping with the pandemic has affected and changed existing healthcare systems worldwide, particularly in terms of their infrastructure, governance and human resources (2). Changes made during the COVID-19 pandemic have been maintained as preventive measures and control strategies to contain the constantly evolving pandemic disease.

During the COVID-19 pandemic, the new norms practiced can be major glitches faced by emergency department (ED) employees (3). The elements of the new norm mainly impacted EDs and involved screening, containing and alerting the nation about infectious diseases. Attempts to prevent the transmission of the disease before it became a pandemic were made. EDs have experienced various outbreaks, such as SARS, H1N1 and COVID-19; therefore, EDs should maintain the changes they have adopted to establish well-prepared systems for coping with future outbreaks (4). This study aimed to determine the elements of the new norm that has introduced changes into the healthcare system, particularly in EDs, during the COVID-19 pandemic. It also aimed to obtain consensus from experts, namely, emergency physicians from across Malaysia, by using the fuzzy Delphi method (FDM). FDM was selected because it is significantly less taxing to the experts in comparison to the traditional Delphi method (DM). Additionally, FDM utilises a more objective quantitative analysis than traditional DM (5, 6). To date, no previous study has explored this topic.

## Methods

**Figure 1:**
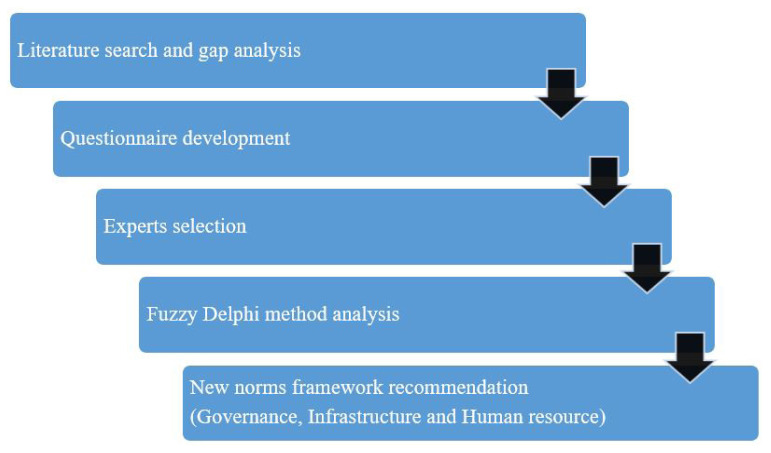
Flow chart of study

FDM was used in this study to effectively acquire expert consensus (Figure 1). This study has two phases as follows:

### Phase 1

Phase 1 involved identifying domains and elements to be assessed and validated by selected experts from a literature review. The domains were selected based on the investigator’s clinical experience at a study centre and after interviewing an experienced emergency physician on the floor. Three major domains were identified: i) infrastructure, ii) governance and iii) human resources. Governance describes the process through which an administration or organisation can consolidate its workflow management by implementing standards, rules and guidelines to achieve objectives simultaneously. Infrastructure refers to treatment facilities. In the COVID-19 context, the designated space includes severe acute respiratory distress isolation rooms, fever centres, negative-pressure airborne isolation rooms and spaces for managing critical and non-critical cases for people under investigation and COVID-19-positive patients. Lastly, human resources include the management of healthcare staff and in the context of this study, they include those working in EDs in Malaysia during the COVID-19 pandemic. FDM elements of assessment are identified based on a rapid review and evidence-based information obtained from sources such as Google Scholar, PubMed Central and the Cochrane Library. Thirty-five items were identified and divided according to their domains. They were sent to Scribendi for English academic editing. The items were then given to a group of three emergency physicians for review and validation based on the research objectives and questions. These emergency physicians were selected based on their expertise and clinical experience. The criteria for selecting an expert for inclusion in the study were as follows:

- The emergency physicians were working in the ED of Hospital Universiti Sains Malaysia.
- The emergency physicians had more than 10 years of working experience in their respective specialties.
- The emergency physicians had experience in managing cases of COVID-19 and severe acute respiratory infection.

The investigator discussed and reviewed all 35 items with the experts individually. Certain elements required modification for content and wording, whereas others must be eliminated or exchanged with other elements for valid reasons as provided by the experts. Finally, the 35 items were modified and prepared in Google Forms. Additional questions inquired about information such as the number of isolation rooms, bed status and current practices.

### Phase 2

Phase 2 of the study involved 15 experts as guided by the FDM, requiring 10–15 respondents. The study was conducted over 3 months, from September 2021 to November 2021. The 15 experts were selected based on their experience in their respective specialties. The criteria for selecting an expert for this phase of this study included the following:

- Emergency physicians working in Malaysia, with more than 5 years of experience in their respective specialties
- Emergency physicians involved in the Ministry of Health Malaysia and Hospital Universiti Sains Malaysia
- Emergency physicians with experience managing COVID-19 and severe acute respiratory infection cases
- Emergency physicians who perform scholarly work on COVID-19 management and outbreaks

The items were distributed to the 15 emergency physicians from across Malaysia through Google Forms link via a text messaging platform, namely, WhatsApp or Telegram. Experts eligible according to the criteria were included and all consented to participate voluntarily. They were asked to assess and declare their level of agreement with the 35 items. These items were the ‘new norms’ introduced as changes in EDs in Malaysia during the COVID-19 pandemic. The participants were given 1 week to respond with an evaluation of the items. Table 1 illustrates the agreement level of seven levels of linguistic variables (Likert scoring) and the fuzzy scale. The fuzzy rating scale was introduced to cope with the imprecision of human thought and experience in measuring attitudes in many fields of study.

The linguistic variables of agreement in the Likert scale format were performed by each expert for each item. Then, these variables were translated into fuzzy number data and analysed in Microsoft Excel FDM software, version 2.0 using FDM as the data analysis technique.

### Fuzzy Delphi Method Implementation Steps

#### Step 1: Selection of Experts

In the selection of experts, satisfactory results can be obtained from small panels of 10–15 individuals from a homogenous group of experts (7).

The definition of expert will vary depending on the context and field of interest to which FDM will be applied. Being an expert in a particular field entails the acquisition of experience, special skills and knowledge of that particular subject. In this context, the selection of experts was as described above.

#### Step 2: Conversion of Linguistic Variables into Triangular Fuzzy Numbers

**Figure 2:**
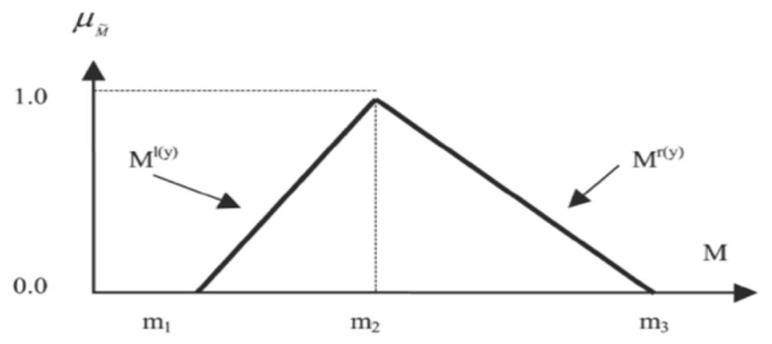
Triangular fuzzy number

The next step involves converting all the linguistic variables into triangular fuzzy numbers. A triangular fuzzy number represents the values of m1, m2 and m3 indicating the minimum, reasonable and maximum values, respectively. Figure 2 illustrates the m1, m2 and m3 values for a triangular fuzzy number. A triangular fuzzy number is used to convert a fuzzy scale (which is similar to a Likert scale). Then, a fuzzy scale is used to convert linguistic variables into fuzzy numbers.

#### Step 3: Identifying the Value of Threshold d

The next step involves identifying the value of threshold *d*. The threshold value is particularly important in the process of identifying the agreement level among the experts. To obtain expert agreement on each item, *d* must not exceed 0.2 (*d* < 0.2) (8). If *d* is less than 0.2 (*d* < 0.2), then all the experts have reached an agreement on an item. Otherwise, a second round should be conducted to consider whether the item is needed or not. This condition is the first requirement that needs to be met for FDM.

The threshold value (*d*) was calculated as follows:


(Equation 1) $$d(\bar{m},\bar{n})=\sqrt{\frac{1}{3}\;[{\left(m1-n1\right)}^{2}+{\left(m2-n2\right)}^{2}+{\left(m3-n3\right)}^{2}]}$$

#### Step 4: Determining Experts’ Agreement Level

The second requirement for the FDM involves determining whether the experts’ agreement is greater than or equal to 75% for each item (9). If the percentage of experts’ agreement is greater than or equal to 75% for an item, then that item can be assumed to achieve expert agreement. The percentage of expert agreement can be calculated by using the following formula:


(Equation 2) $$\text{Percentage of expert agreement}=\frac{Number\;of\;items\;d<0.2}{Total\;items}\times 100\%$$

#### Step 5: Consensus of Experts for Receiving an Item

The third criterion required for FDM is the α-cut. If α-cut is greater than or equal to 0.5 (10) then the item will be accepted as it shows the consensus of experts on receiving the item. The calculation and determination of the fuzzy values are determined as follows:


(Equation 3) $$\begin{array}{ll} \text{i.} & A=1/3*(\text{m}1+\text{m}2+\text{m}3,\text{or},\\ \text{ii.} & A=1/4*(\text{m}1+2\text{m}2+\text{m}3),\;\text{or},\\ \text{iii.} & A=1/6*(\text{m}1+4\text{m}2+\text{m}3). \end{array}$$

If *A* is more than the α-cut value of 0.5, then the item will be accepted as it shows the consensus of the expert on retaining the item.

#### Step 6: Ranking an Item

This step ranks the items into sub-phases. The ranking steps are determined by selecting an item based on its defuzzification value obtained from experts’ agreement. The item with the most important ranking in the model has the highest value.

## Results

Thirty-five items were identified for the new norms from a literature search of all three domains (governance, infrastructure and human resources) (Table 2). All the items were initially scored based on linguistic variables (Likert scale scoring) and these scores were converted into fuzzy numbers. The fuzzy numbers were recorded, and average m1, m2 and m3 values were calculated. The higher the fuzzy number value, the higher the level of agreement. The value of the threshold *d* was obtained by Equation 1. Nine of the 35 items did not meet the first requirement for FDM as the threshold value *d* must be less than 0.2 (*d* < 0.2).

In the second requirement for FDM, 20 of the 35 items had an expert consensus of > 75%. For the third requirement, only one item did not meet the criterion for an average fuzzy number (*A* value) of > 0.5. Finally, in this study, only 25 items met all three requirements of FDM and were therefore retained, whereas the remaining 10 items were discarded. The final step was defuzzification performed during the data analysis using the FDM technique. The defuzzification step determines the ranking of each item. Supplementary Table 1 summarises all three domains after analysing the findings of FDM for the new norm elements in the EDs in Malaysia during the COVID-19 pandemic. Approximately 30% (*n* = 5), 18% (*n* = 2) and 25% (*n* = 2) of the items were finally discarded for governance, infrastructure and human resource domains, respectively. Supplementary Table 2 shows the detailed descriptions of each item and their ranking of acceptance based on the FDM criteria (Supplementary Tables 1 and 2 files)

## Discussion

This study obtains experts’ consensus on the findings of the new norm items in EDs in Malaysia during the COVID-19 pandemic. The findings of this study showed that the retained items should form the new norms in EDs during the COVID-19 pandemic as these items have all obtained expert consensus. The retained items can be used to develop guidelines as the FDM findings involved all experts’ opinions. Certain experts have the authority to develop protocols in the field, whereas others are more experienced and knowledgeable.

In the area of governance, this study obtained expert consensus on 11 items to be retained, with the remaining five to be discarded. Among the top three rankings of the retained items, the experts have reached an agreement to establish ED guidelines for COVID-19 preparedness and response strategy to be applied at the hospital level. This study also found that the operational planning and development of emergency response protocols will better prepare EDs for the pandemic and any future outbreaks (11). A pandemic crisis such as COVID-19 requires a preparedness plan to manage disasters in the short and long term (12). Furthermore, the top-ranked item includes the use of standard personal protective equipment (PPE) for aerosol-generating procedures (such as intubation). The PPE should include a disposable isolation gown suit, a face shield, an N95 mask, boot covers and gloves. Although a powered air-purifying respirator provides greater protection than the standard PPE, the standard PPE for aerosol-generating procedures is top-ranked by experts due to several reasons, such as the availability of the device and cost-effectiveness. According to the Centers for Disease Control and Prevent, respirators with N95 filters or higher are used for all aerosol-generating procedures, consistent with the study’s findings (13). The third-ranked item involves establishing standard operational procedures for the training and clinical management of COVID-19 and updating them regularly. Based on expert consensus, the discarded elements include the use of an aerosol box to minimise the spread of COVID-19 when performing aerosol-generating procedures (such as intubation) and an isolation pod to transfer COVID-19 patients and the development of a teleconsultation helpline service. Evidence for the effectiveness of the aerosol box or any barriers is currently insufficient and suggests that they may bring additional risks (14). However, teleconsultation has gained significant visibility in many countries, particularly in Western countries. Currently, it has been used in previous epidemics, such as Ebola and SARS. Teleconsultation aims to prevent disease transmission, reduce patient volume and load, and provide psychosocial support services. Thus, health authorities in several countries have already implemented telemedicine user guidelines to provide support during the pandemic (15). Implementing teleconsultation would be helpful in the clinical setting, particularly during the pandemic (16). Based on this study, we should consider adopting teleconsultation in EDs.

Eleven items for infrastructure were initially extracted from the literature search. Following the FDM analysis, nine items were retained, whereas two were not accepted by the expert consensus. The items retained include designating respiratory and non-respiratory zones in EDs with separate entrance and exit points, an isolation room with or without negative pressure for COVID-19 patients with separate entrance and exit points, PPE donning/doffing area in EDs, predetermined transport routes to transfer COVID-19 patients to minimise exposure, and other elements as stated in Supplementary Table 1. Some of the emergency physicians stated that their EDs had a minimum of 1**–**5 isolation rooms without negative pressure or a bay with a minimum 5–60-bed capacity. For the negative-pressure isolation rooms, a minimum of 1–5 rooms were required with a 1–12-bed capacity. Most EDS in Malaysia are not equipped with negative-pressure isolation rooms due to their high cost. Building negative-pressure isolation rooms is one of the ideal ways of controlling the spread of infectious respiratory diseases to prevent the transmission of airborne infections (17). In Middle Eastern countries, health ministries have mandated certain hospitals to designate a separate respiratory zone after the 2012 Middle East respiratory syndrome coronavirus (MERS-CoV) outbreak to prevent mixing infected patients (18, 19). In the future, hospitals with more treatment spaces designated for infectious diseases in the ED should be built. For the discarded elements, cabins or tents with and without a negative-pressure system for fever centre/acute respiratory influenza-like illnesses designated outside of EDs were unfavourable among the experts. The lack of treatment spaces in EDs leads to restructuring and reconverting other available spaces into COVID-19 treatment areas. In other places, patients are triaged and screened based on presenting complaints and risk stratification of COVID-19 into the respiratory and non-respiratory zones (20).

The human resources group had eight items: three were discarded and the remaining five were retained. After the defuzzification step, the top three priority items were regular training of healthcare workers on performing nasopharyngeal swabs and PPE donning/doffing, providing hospital scrubs to all healthcare workers in the respiratory zone, and conducting close contact tracing by the ED team of suspected and confirmed COVID-19 cases among its healthcare workers. Nasopharyngeal or oropharyngeal swabs remain the gold standard for diagnosing COVID-19, and improper techniques for collecting COVID-19 samples can lead to false-negative COVID-19 results. Therefore, training sessions in swabbing techniques improve sampling techniques, knowledge and confidence (21). PPE training is essential to protecting healthcare workers, especially during pandemics. A recent study investigated whether the frequent use of incorrect steps when donning and doffing PPE was a risk for contamination. It revealed that suboptimal training and education are significant contributing factors (22). Another recent study showed that contamination while removing PPE is significant. Hence, preventive measures, such as intensive and repeated training, interactive audio-visuals, and the assistance of trained personnel during donning and doffing, should be conducted frequently (23). Providing hospital scrubs to all healthcare workers in the respiratory zone can prevent disease transmission among them (24). The other retained items for the human resource management domain are interdepartmental recruitment and rotation. They will solve the issues of work scheduling, redeployment and staff shortages. Frequent training programmes and strict standard operating procedures on the use of PPE will ensure adherence to infection control and staff safety. Health surveillance mechanisms must be established to monitor and ensure mental and physical well-being throughout the pandemic.

Different healthcare systems may have different priorities for the items to be included, depending on local settings or as suited to their particular needs. Each ED must respond in various ways depending on the features of infectious diseases and the surge of cases. At the early stage, whether a variant can be super-spreading in a short period, such as the COVID-19 pandemic, cannot be determined. The implications of this pandemic have introduced drastic changes to EDs. The changes made during the COVID-19 pandemic have been maintained as preventive measures and control strategies to contain the constantly evolving pandemic disease.

Thus, FDM can be used to develop guidelines, standard protocols and optimal workflows by achieving consensus among experts. FDM is also advantageous because it ranks items, indicating the relative importance of the selected elements, whereas unfit elements are discarded based on expert consensus. Therefore, one may select the best items suited for one’s needs based on the ranking priority as one size may not fit all. Selecting experts who have had significant experience with the intervention approach and having a balanced number of experts with a similar job scope will help to improve the validity of these findings. FDM has been, and will continue to be, an important data collection methodology with various applications and uses for people who aim to gather expert information on a particular subject (25). Expert opinions and involvement in various dimensions positively impact the formation of guidelines.

### Limitation of Study

A weakness in the FDM includes the constant reminders needed before the experts can provide their responses. At the same time, the experts’ views may have been limited to their perspectives and experience and thus may not have represented the views of the entire fraternity. In addition, no comments were given by the experts to explain their low scale scores for certain elements as FDM is based on the Likert-type scale scoring without any request to explain the reason for the answer.

## Conclusion

The impact of COVID-19 has led to the introduction of extraordinarily new norms and has reshaped our daily workflow; no one can predict how long a pandemic will last. The current standards of practice, which were previously rare, are being adapted well. The findings of this study are based on the expert consensus. Twenty-five items within the domains of governance, infrastructure and human resource management were accepted by expert consensus and met all three requirements of FDM. The three domains cover strict adherence to infection control and screening mechanisms. The experts also agreed that ensuring staff well-being during outbreaks is a crucial item that was retained within the domain of human resource management. Hence, this consensus would provide helpful guidelines for implementation in EDs when managing the COVID-19 pandemic or other newly emerging infectious diseases.

## Figures and Tables

**Table 1:** Level of agreement and fuzzy scale (7 points)

Linguistic variables	Fuzzy scale
Not at all important	(0.0, 0.0, 0.1)
Low important	(0.0, 0.1, 0.3)
Slightly important	(0.1, 0.3, 0.5)
Somewhat important	(0.3, 0.5, 0.7)
Moderately important	(0.5, 0.7, 0.9)
Very important	(0.7, 0.9, 1.0)
Extremely important	(0.9, 1.0, 1.0)

**Table 2:** Initial items obtained from the literature review before FDM analysis

Domain	New norm element/item description (NN)

Governance (G)	
NNG-1	Usage of proper personal protective equipment (PPE) is compulsory during the triaging process.
NNG-2	The creation of a screening process (questionnaire) to aid in clinical triaging of COVID-19 and facilitating zone disposition based on PPE requirements.
NNG-3a	Conducting a questionnaire-based self-reporting screening process of COVID-19 on patients and family members or caretakers before they enter the ED.
NNG-3b	Screening patients and family members or caretakers before they enter the ED using COVID-19 rapid test kits (RTK).
NNG-3c	Screening patients and family members or caretakers before they enter the ED using a questionnaire-based self-reporting and COVID-19 RTK.
NNG-4	Screening for COVID-19 symptoms and measuring temperature of all visitors at the ED entrance.
NNG-5	Healthcare workers performing aerosol-generating procedures (such as intubation) must use an aerosol box to minimise the spread of COVID-19.
NNG-6	Standard PPE worn by healthcare workers when attending to patients without COVID-19.
NNG-7a	PPE: A facial shield or eye protection (goggles), a surgical mask and gloves The standard PPE for aerosol-generating procedures (such as intubation) includes a powered air-purifying respirator (PAPR), N95 mask, boot covers and gloves.
NNG-7b	The standard PPE for aerosol-generating procedures (such as intubation) includes a disposable isolation gown suit (coveralls suit), a face shield, an N95 mask, boot covers, and gloves.
NNG-8	A standard intubation checklist for suspected and confirmed patients with COVID-19.
NNG-9	Using an isolation pod (ISOPOD) to transfer suspected and confirmed patients with COVID-19.
NNG-10	Developing teleconsultation helpline services at the ED to facilitate the screening and triaging process of suspected, probable or confirmed patients with COVID-19.
NNG-11	A computerised system or manual clerking sheet template with the checklist method to augment the speed and efficiency of management.
NNG-12	Establishing standard operational protocols or guidelines on training and clinical management of COVID-19 and updating it promptly.
NNG-13	Developing a guideline for the ED on COVID-19 preparedness and response strategy at the hospital level.

**Infrastructure (I)**	

NNI-14a	Designated negative pressure system cabins or tents for fever centre/acute respiratory influenza-like illness outside of the ED.
NNI-14b	Designated cabins or tents without a negative pressure system for fever centre/acute respiratory influenza-like illness outside of the ED.
NNI-15	Restructuring a respiratory and non-respiratory zone within the ED with separate access and exit points.
NNI-16a	Establishing a negative pressure isolation room for suspected, probable or confirmed COVID-19 cases with separate access and exit points.
NNI-16b	Establishing an isolation room without a negative pressure system for suspected, probable or confirmed COVID-19 cases with separate access and exit points.
NNI-17	Establishing a separate resuscitation area for non-COVID-19 cases with separate access and exit points.
NNI-18	A designated PPE donning/doffing area in the ED.
NNI-19	A designated portable X-ray room in the ED.
NNI-20	The isolation area should have dedicated washrooms, hand-washing stations and trash bins.
NNI-21	Creating predetermined transport routes to transfer patients with COVID-19 to minimise exposure.
NNI-22	During access block, establish an observation ward for suspected, probable, or confirmed COVID-19 and non-COVID-19 cases.

**Human resources (HR)**	

NNHR-23	Setting up a COVID-19 team and rotating the team occasionally to minimise the risk of COVID-19 transmission.
NNHR-24	Interdepartmental recruitment of a COVID-19 team within the hospital to manage suspected, probable, or confirmed patients with COVID-19 in the ED.
NNHR-25	Frequent training of healthcare workers on performing nasopharyngeal swabs, donning and doffing PPE.
NNHR-26	To ensure safety, high-risk healthcare workers (workers with pre-existing comorbidities and pregnancy) should work in a non-COVID-19 zone.
NNHR-27	Active surveillance of COVID-19 risk in emergency healthcare workers upon arrival at work.
NNHR-28	Active mental health surveillance on emergency healthcare workers during the pandemic.
NNHR-29	Providing hospital scrubs to all healthcare workers working in the respiratory zone.
NNHR-30	The ED team conducts close contact tracing of suspected or confirmed COVID-19 cases among healthcare workers.

## Appendix

**Supplementary Table 1:** Summary of all three domains after Fuzzy Delphi analysis findings for the new norm elements in ED in Malaysia during the COVID-19 pandemic

No.	Items/Elements	Threshold value, *D* < 0.2	Percentage of expert consensus, %	Average of fuzzy numbers (*A* value)	Ranking	Verdict
Domain: Governance						
1	NNG-1	0.094	93.3%	0.909	6	Retained
2	NNG-2	0.111	86.7%	0.911	5	Retained
3	NNG-3a	0.138	93.3%	0.898	9	Retained
4	NNG-3b	0.425	33.33%	0.613	15	Discarded
5	NNG-3c	0.383	40.00%	0.638	14	Discarded
6	NNG-4	0.094	93.33%	0.909	6	Retained
7	NNG-5	0.377	40.00%	0.371	16	Discarded
8	NNG-6	0.068	100.00%	0.933	3	Retained
9	NNG-7a	0.252	86.67%	0.818	11	Retained
10	NNG-7b	0.060	100.00%	0.940	1	Retained
11	NNG-8	0.094	93.33%	0.909	6	Retained
12	NNG-9	0.402	46.67%	0.642	13	Discarded
13	NNG-10	0.290	60.00%	0.742	12	Discarded
14	NNG-11	0.094	100.00%	0.878	10	Retained
15	NNG-12	0.068	100.00%	0.933	3	Retained
16	NNG-13	0.060	100.00%	0.940	1	Retained
DOMAIN: INFRASTRUCTURE						
1	NNI-14a	0.321	53.3%	0.738	10	Discarded
2	NNI-14b	0.410	40.0%	0.716	11	Discarded
3	NNI-15	0.116	93.3%	0.909	4	Retained
4	NNI-16a	0.155	93.33%	0.873	6	Retained
5	NNI-16b	0.186	93.33%	0.829	9	Retained
6	NNI-17	0.244	86.67%	0.842	8	Retained
7	NNI-18	0.089	93.33%	0.922	1	Retained
8	NNI-19	0.183	93.33%	0.860	7	Retained
9	NNI-20	0.076	100.00%	0.920	2	Retained
10	NNI-21	0.076	100.00%	0.913	3	Retained
11	NNI-22	0.131	93.33%	0.878	5	Retained
DOMAIN: HUMAN RESOURCE						
1	NNHR-23	0.409	20.0%	0.647	8	Discarded
2	NNHR-24	0.291	66.7%	0.704	6	Discarded
3	NNHR-25	0.087	100.0%	0.896	1	Retained
4	NNHR-26	0.373	40.00%	0.662	7	Discarded
5	NNHR-27	0.197	93.33%	0.842	4	Retained
6	NNHR-28	0.145	93.33%	0.829	5	Retained
7	NNHR-29	0.173	93.33%	0.878	2	Retained
8	NNHR-30	0.119	93.33%	0.864	3	Retained

Notes: Threshold value (*d*) < 0.2 (three decimal points is accepted); Percentage expert consensus agreement > 75%; Average fuzzy value (*A* value ) > 0.5; All three must be fulfilled for the elements to be retained

**Supplementary Table 2:** Summary of all three domains after Fuzzy Delphi analysis findings for the new norm elements in ED in Malaysia during the COVID-19 pandemic

Domain	New norm element/item description (NN)	Verdict	Ranking

Governance (G)			
NNG-1	Usage of proper Personal Protective Equipment (PPE) is compulsory during the triaging process.	Retained	6
NNG-2	The creation of a screening process (questionnaire) to aid in clinical triaging of COVID-19 and facilitating zone disposition based on PPE requirements.	Retained	5
NNG-3a	Conducting a questionnaire-based self-reporting screening process of COVID-19 on patients and family members or caretakers before they enter the emergency department (ED).	Retained	9
NNG-3b	Screening patients and family members or caretakers before they enter the ED using COVID-19 rapid test kits (RTK).	Discarded	15
NNG-3c	Screening patients and family members or caretakers before they enter the ED using a questionnaire-based self-reporting and COVID-19 rapid test kits (RTK).	Discarded	14
NNG-4	Screening for COVID-19 symptoms and measuring the temperature of all visitors at the ED entrance.	Retained	6
NNG-5	Healthcare workers performing aerosol-generating procedures (such as intubation) must use an aerosol box to minimise the spread of COVID-19.	Discarded	16
NNG-6	Standard PPE worn by healthcare workers when attending to patients without COVID -19. PPE: A facial shield or eye protection (goggles), a surgical mask and gloves.	Retained	3
NNG-7a	The standard PPE for aerosol-generating procedures (such as intubation) includes a powered air-purifying respirator (PAPR), N95 mask, boot covers, and gloves.	Retained	11
NNG-7b	The standard PPE for aerosol-generating procedures (such as intubation) includes a disposable isolation gown suit (coveralls suit), a face shield, an N95 mask, boot covers, and gloves.	Retained	1
NNG-8	A standard intubation checklist for suspected and confirmed patients with COVID-19.	Retained	6
NNG-9	Using an isolation pod (ISOPOD) to transfer suspected and confirmed patients with COVID-19.	Discarded	13
NNG-10	Developing teleconsultation helpline services at the ED to facilitate the screening and triaging process of suspected, probable, or confirmed patients with COVID-19.	Discarded	12
NNG-11	A computerised system or manual clerking sheet template with the checklist method augments the speed and efficiency of management.	Retained	10
NNG-12	Establishing standard operational protocols or guidelines on training and clinical management of COVID-19 and updating it promptly.	Retained	3
NNG-13	Developing a guideline for the ED on COVID-19 preparedness and response strategy at the hospital level.	Retained	1

**Infrastructure**			

NNI-14a	Designated negative pressure system cabins or tents for fever centre/acute respiratory influenza-like illness outside of the ED.	Discarded	10
NNI-14b	Designated cabins or tents without a negative pressure system for fever centre/acute respiratory influenza-like illness outside of the ED.	Discarded	11
NNI-15	Restructuring a respiratory and non-respiratory zone within the ED with separate access and exit points.	Retained	4
NNI-16a	Establishing a negative pressure isolation room for suspected, probable, or confirmed COVID-19 cases with separate access and exit points.	Retained	6
NNI-16b	Establishing an isolation room without a negative pressure system for suspected, probable, or confirmed COVID-19 cases with separate access and exit points.	Retained	9
NNI-17	Establishing a separate resuscitation area for non-COVID-19 cases with separate access and exit points.	Retained	8
NNI-18	A designated PPE donning/ doffing area in the ED	Retained	1
NNI-19	A designated portable X-ray room in the ED.	Retained	7
NNI-20	The isolation area should have dedicated washrooms, hand-washing stations, and trash bins.	Retained	2
NNI-21	Creating predetermined transport routes to transfer patients with COVID-19 to minimise exposure.	Retained	3
NNI-22	During access block, establish an observation ward for suspected, probable, or confirmed COVID-19 and non-COVID-19 cases.	Retained	5

**Human resources (HR)**			

NNHR-23	Setting up a COVID-19 team and rotating the team occasionally to minimise the risk of COVID-19 transmission.	Discarded	8
NNHR-24	Interdepartmental recruitment of a COVID-19 team within the hospital to manage suspected, probable, or confirmed patients with COVID-19 in the ED.	Discarded	6
NNHR-25	Frequent training of healthcare workers on performing nasopharyngeal swabs, donning and doffing PPE.	Retained	1
NNHR-26	To ensure safety, high-risk healthcare workers (workers with pre-existing comorbidities and pregnancy) should work in a non-COVID-19 zone.	Discarded	7
NNHR-27	Active surveillance of COVID-19 risk in emergency healthcare workers upon arrival at work.	Retained	4
NNHR-28	Active mental health surveillance on emergency healthcare workers during the pandemic.	Retained	5
NNHR-29	Providing hospital scrubs to all healthcare workers working in the respiratory zone.	Retained	2
NNHR-30	The ED team conducts close contact tracing of suspected or confirmed COVID-19 cases among healthcare workers.	Retained	3
